# Is there any advantage to combined trastuzumab and chemotherapy in perioperative setting her 2neu positive localized gastric adenocarcinoma?

**DOI:** 10.1186/1477-7819-9-112

**Published:** 2011-09-28

**Authors:** Yassir Sbitti, Ismail Essaidi l, Adil Debbagh, Habiba Kadiri, Mohamed Oukabli, Yassine Moussaid, Khaoula Slimani, Mohamed Fetohi, Hakim Elkaoui, Abderrahmane Albouzidi, Mohamed Mahi, Abdelmounaim Ait Ali, Mohamed Ichou, Hassan Errihani

**Affiliations:** 1 Department of Medical Oncology, University Military Hospital; Rabat, 10000, Morocco; 2 Department of Pathology Diagnostic Center, Rabat, 10000, Morocco; 3 Department of Pathology, University Military Hospital of instruction; Rabat, 10000, Morocco; 4 Department of Surgery, University Military Hospital of instruction; Rabat, 10000, Morocco; 5 Department of Radiology, University Military Hospital of instruction; Rabat, 10000, Morocco

**Keywords:** trastuzumab, chemotherapy, perioperative, gastric adenocarcinoma, resection

## Abstract

We report here a 44-year-old Moroccan man with resectable gastric adenocarcinoma with overexpression of human epidermal growth factor receptor 2 (HER2) by immunohistochemistry who was treated with trastuzumab in combination with chemotherapy in perioperative setting. He received 3 cycles of neoadjuvant chemotherapy consisting of trastuzumab, oxaliplatin, and capecitabine. Afterwards, he received total gastrectomy with extended D2 lymphadenectomy without spleno-pancreatectomy. A pathologic complete response was obtained with a combination of trastuzumab and oxaliplatin and capecitabine. He received 3 more cycles of trastuzumab containing regimen postoperatively.

We conclude that resectable gastric carcinoma with overexpression of the c-erbB-2 protein should ideally be managed with perioperative combination of trastuzumab with chemotherapy. Further research to evaluate trastuzumab in combination with chemotherapy regimens in the perioperative and adjuvant setting is urgently needed.

## Background

Gastric cancer is the second largest cause of cancer associated death world-wide. Surgery remains the mainstay of treatment for the resectable cancer. However with the noted high frequency of loco regional and distant recurrences and relatively low 5-year survival for symptomatic Stage II-III and Stage IV cancer (20-50% and 5-10%, respectively), there has been a need to develop more effective peri-operative and adjuvant therapies for Stage II-IV disease [1] and in some countries with a high incidence of gastric cancer (such as Japan) screening programs have been established for the detection of Stage I resectable disease which has a 90% chance of 5-year survival [1]. Perioperative chemotherapy has been shown to cause tumor down staging and improve survival in patients with resectable gastric cancer [2]. Response to neoadjuvant treatment is the most important predictor of survival after curative resection of gastric cancer [3,4]. More recently several novel approaches based on molecular targeting have also been attempted including the use of anti-VEGF [5], EGFR [6] or HER2 [7] monoclonal antibodies combined with chemotherapy. In this case report, we describe a case of neoadjuvant chemotherapy with trastuzumab-containing regimen in gastric cancer. We discuss histopathological effect and review the literatures.

## Case presentation

**Figure 1:**
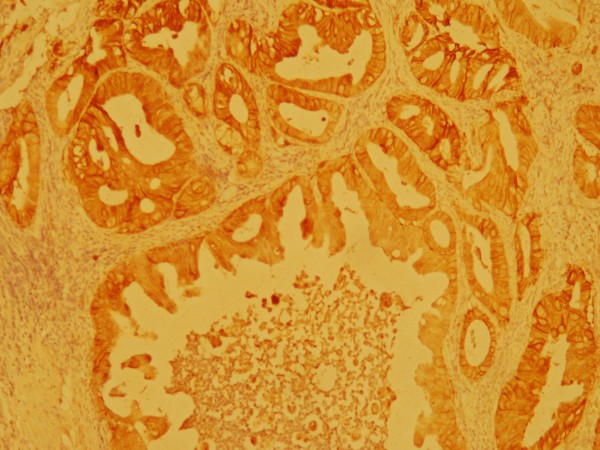
**Immunohistochemical study of HER2 protein in biopsied specimen before chemotherapy**. The well differentiated adenocarcinoma cells infiltrated the gastric sub mucosa and Strong complete, basolateral membranous reactivity in > 80% of the tumor cells in favor of over expressed HER2 (3+ by HercepTest) on the cell membrane (immunoperoxidase stain, 100×).

**Figure 2:**
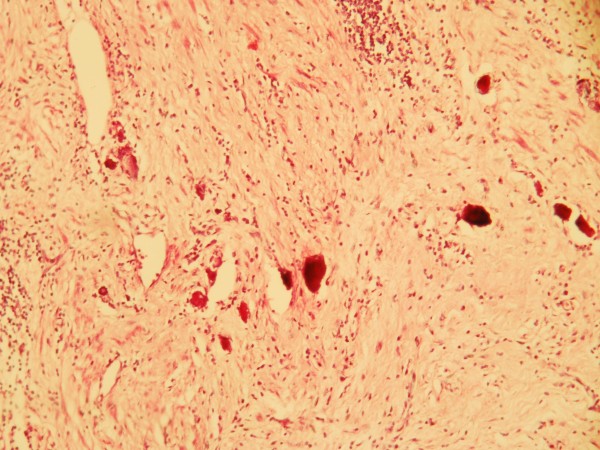
**Microscopic finding of the resected specimen after chemotherapy**. No residual adenocarcinoma was found in the original ulcerated adenocarcinoma site on lesser curvature. Instead, it was completely replaced by dense fibrous tissue (hematoxylin eosin stain, 100×).

At the end of April 2010 a healthy 44 years Old Moroccan male without medical history was admitted at our institution for incoercible vomiting with moelena. He underwent oesophageogastroduodenoscopy witch showed a 3-cm gastric polypoides lesions on the lesser curvature proximal to angularis. Specimen Gastric biopsy revealed an infiltrating well differentiated adenocarcinoma. Tumor analysis for human epidermal growth factor receptor 2 (HER2) was performed by HercepTest ventana indicating a Strong complete, basolateral membranous reactivity in > 80% of the tumor cells in favor of 3+ immunohistochemistry (IHC) staining (Figure 1). Staging workups, including computed tomography (CT) scan of chest, abdomen and pelvis showed a circumferential and irregular thickening fundic area arriving in contact with body pancreas without infiltration sign without loco regional lymph node. Triphasic (CT) revealed a lesion involving segments 4, 5 and 7 of the liver. It was centrally hypodense with peripheral enhancement in the arterial phase suggesting an angiomatose lesions or secondary localizations. Positron Emission Tomography-CT scan was not available. In front of this doubt about hepatic lesions, endoscopic ultrasound was not retained and platinum based chemotherapy regimen including Capecitabine (2000 mg/m^2^/j) po bid on day 1 to day 14 plus oxaliplatin (130 mg/m^2^/j) on day 1 were given every 3 weeks. Trastuzumab (intravenously, 8 mg/kg loading dose, then 6 mg/kg on days 1-21 of every cycle) was started at the end of MAY 2010 and administered concommittally with chemotherapy for three cycles. Post CT scan evaluation showed a gastric partial response with stability of hepatic lesions. Hepatic Magnetic Resonance Imagery with diffusion technique objective of atypical hemangioma lesion. Therapeutic strategy was reconsidered and total gastrectomy with extended D1.5 lymph node dissections, Roux-en-Y esophagojejunostomygastric surgery was practiced in August 2010. Prior to surgical resection, laparoscopy revealed no evidence of peritoneal carcinomatosis or metastatic implants. Pathological examination of the surgical specimen indicated no residual adenocarcinoma but scar on lesser curvature with fibrosis extending into muscularis propria (Figure 2). There were no tumor identified in 24 perigastric lymph nodes and 2 lymph nodes from porta hepatis. He recovered uneventfully after surgery, and received 3 more cycles of chemotherapy consisting of trastuzumab, oxaliplatine. After gastrectomy, our patient presented loss of appetite, and dietary problems. Most important advice (to eat small, frequent meals) following a gastrectomy was proposed. Oral Capecitabine was substituted by intravenous perfusion of 5FU for 96 hours. Last cycle of treatment was given in November 2010. He has remained free of disease after completion of chemotherapy. We have monitored our patient's cardiac function with periodic echocardiogram evaluation, and find no evidence of cardiac failure. Most common toxicities were (grade 1) neuropathy and hand-foot syndrome. Currently, the patient is under monitoring. He underwent periodic follow-up with CT scan. He received intramuscular supplementation of vitamin B12. He is in good health without recurrence for 15 months.

## Conclusion

HER2 protein over expression by immunohistochemistry (IHC) and/or erB2 gene amplification by in situ hybridization was detected in 4-28% of gastric or gastro-oesophageal junction cancers (GOJ) [8]. HER2/neu positivity rates have been reported to be more frequent in intestinal type gastric cancer (21.5%) than in diffuse gastric cancer (2%) or mixed types (5%) [9]. Most studies have shown that HER2-overexpressing gastric cancers were worse prognosis and have been shown to be an independent prognostic factor [10]. Trastuzumab is a humanized monoclonal antibody directed against HER2 with known efficacy in patients with HER2+ early or metastatic breast cancer. Results from the largest study to date (ToGA trial) evaluating the addition of trastuzumab to chemotherapy in HER2-positive advanced gastric cancer (AGC) were reported at the 2009 American Society Clinical Oncology (ASCO) meeting [11]. The Trastuzumab for Gastric Cancer (ToGA) trial is the first randomized Phase III trial providing prospective information on HER2-positivity rates in AGC. The trial enrolled 3,883 patients from 24 countries. A HER2-scoring system modified from the protocol in breast cancer was used: a score of IHC 3+ and/or FISH positive was defined as HER2 positive. The modified HER2-scoring system showed concordance between IHC and FISH results of 87.5%. In breast cancer most IHC 0/1 samples are FISH negative but, in the ToGA cohort, the frequency of IHC 0/1 samples testing FISH positive was almost as high as IHC 2/FISH-positive samples (23% vs. 26%). The study reported an overall HER2-positivity rate of 22.1% evaluated from 3807 patients. In the ToGA trial, patients with HER2-positive gastroesophageal and gastric adenocarcinoma (locally advanced, recurrent, or metastatic) were randomized to receive Trastuzumab plus chemotherapy (5-fluorouracil or capecitabine and cisplatin) q3w for 6 cycles or chemotherapy alone. The primary end point was overall survival (OS); secondary end points included overall response rate (ORR), progression-free survival, time to progression, duration of response, and safety. Median OS was significantly improved with Trastuzumab plus chemotherapy compared to chemotherapy alone: 13.5 vs. 11.1 months, respectively (p = 0.0048; HR 0.74; 95% CI 0.60, 0.91). (ORR) was 47.3% in the Trastuzumab plus chemotherapy arm and 34.5% in the chemotherapy arm (p = 0.0017). This first randomized trial investigating anti-HER2 therapy in AGC showed that Trastuzumab plus chemotherapy is superior to chemotherapy alone. The OS benefit indicates that trastuzumab is a new, effective, and well-tolerated treatment for HER2-positive AGC. The benefit was even greater in the subgroup with HER2 overexpression (16% of the screened population) as defined by IHC3+ or IHC2+ confirmed by positive ISH test [12]. Trastuzumab plus FP chemotherapy has become the standard treatment for patients with HER2+ non-pretreated metastatic adenocarcinoma of the stomach or GOJ cancer. The MAGIC trial showed that patients treated with Perioperative epirubicin, cisplatin, and 5-fluorouracil had significantly higher overall survival compared to patients treated with surgery alone (5-year survival: 36% for chemotherapy plus surgery vs. 23% for surgery). At the time of surgery, the patients receiving preoperative chemotherapy had significantly smaller tumor size and lower stage. However, there was no pathological complete response in patients receiving preoperative ECF in this study [2]. The infusional 5-FU in the ECF regimen is given continuously through a venous access device, and is associated with inconvenience and higher incidence of thrombosis and infection. Furthermore, cisplatin can cause nephrotoxicity, ototoxicity, and severe emesis. The benefit for preoperative chemotherapy was also noted in a French multicenter trial in which 224 patients with potentially resectable stage II or greater adenocarcinoma of the stomach (n = 55), GE junction (n = 144) or distal esophagus (n = 25) were randomly assigned to two to three cycles of preoperative chemotherapy (infusional 5-FU 800 mg/m2 daily for five days plus cisplatin 100 mg/m2 on day 1 or 2, every four weeks) or surgery alone. In a Final report, patients undergoing neoadjuvant chemotherapy were significantly more likely to undergo R0 (microscopically complete) resection (87 versus 74 percent), and there was a statistically insignificant trend toward fewer pT3/4 (58 versus 68 percent) and fewer node-positive tumors (67 versus 80 percent) that favored this group as well.

With a median 5.7-year follow-up, neoadjuvant chemotherapy was associated with a significant 35 percent reduction in the risk of disease recurrence (five-year disease-free survival 34 versus 21 percent) and a significant, 31 percent lower risk of death (five-year survival 38 versus 24 percent) [13]. REAL-2, a randomized study in patients with advanced gastroesophageal cancer using two-by-two design, has shown 5-FU can be replaced by capecitabine and cisplatin by oxaliplatin in the regimen of ECF without affecting the efficacy [14]. Other studies also show that oxaliplatin can be substituted for cisplatin [15] and Capecitabine for 5-FU in chemotherapy doublets [16], preserving efficacy and offering some toxicity benefits. A recent meta-analysis has shown that Capecitabine is superior to infused 5-FU for OS within doublet and triplet regimes for advanced gastric cancer [17]. Initially our patient was considered metastatic at baseline and Trastuzumab based regimen was received as standard treatment. Substitution of oxaliplatin and Capecitabine was based on increased tolerance of without efficacy loss in advanced setting. Hepatic hemangioma lesion showed in magnetic resonance imagery led us to reconsider disease stage and propose curative therapeutic strategy. He received total gastrectomy with extended D1.5 lymph node dissections showing pathological complete response significantly influencing relapse-free survival, overall survival. This information was observed in locally invasive breast cancer. The results of 3 large phase III trials (the M. D. Anderson Cancer Center neoadjuvant trastuzumab trial, the Neoadjuvant Herceptin [NOAH] trial, and the German Breast Group/Gynecologic Oncology Study Group "GeparQuattro" trial) demonstrated that, compared with chemotherapy alone, neoadjuvant trastuzumab plus chemotherapy significantly increased pathologic complete response rates to as high as 65%, improvements in disease-free, event-free, and overall survival [17-19]. However, the question which arose was the duration of trastuzumab. it's necessary to manage for 12 months by extrapolation from adjuvant breast cancer or to be satisfied with 6 cycles in totality?. The answer to this question requires a large randomized phase III or II study. Our case illustrates the case of pathological complete response after neoadjuvant chemotherapy with trastuzumab-containing regimen in a patient with locally gastric cancer over expressing HER2. The use of oxaliplatin and capecitabine in combination with trastuzumab in this setting remains experimental, and ideally should be considered only in the context of a clinical trial. Therefore, the role of trastuzumab as a part of perioperative therapy is worth further investigation. Multidisciplinary evaluation plays a crucial role in the management of these patients.

## Consent

Written informed consent was obtained from the patient for publication of this case report and any accompanying images. A copy of the written consent is available for review by the Editor-in-Chief of this journal.

## List of abbreviations

HER2: Human Epidermal Growth Factor Receptor 2; GOJ: Gastro-Esophageal Junction; CT: computed tomography; IHC: immunohistochemistry; AGC: Advanced Gastric Cancer; ASCO: American Society Clinical Oncology; ToGA: Trastuzumab for Gastric Cancer; OS: Overall Survival; ORR: Overall Response Rate.

## Competing interests

The authors declare that they have no competing interests.

## Authors' contributions

SY designed and wrote the paper. MM performed radiologic workup (CT and MRI). AA, HE performed surgery. HK, MO and AA provided pathological diagnosis and evaluation. SY, EI, AD, YM, KS, MF participate in medical treatment. MI and HE designed the paper. All authors read and approved the final manuscript.
